# Horner's Syndrome due to a Spontaneous Internal Carotid Artery Dissection after Deep Sea Scuba Diving

**DOI:** 10.1155/2016/5162869

**Published:** 2016-07-20

**Authors:** Jose Enrique Alonso Formento, Jose Luis Fernández Reyes, Blanca Mar Envid Lázaro, Teresa Fernández Letamendi, Ryth Yeste Martín, Francisco José Jódar Morente

**Affiliations:** ^1^Emergency Medicine Unit, Miguel Servet Hospital, 50009 Zaragoza, Spain; ^2^Internal Medicine Unit, Hospital Médico-Quirúrgico del Complejo Hospitalario de Jaén, 23007 Jaén, Spain

## Abstract

Internal carotid artery dissection (ICAD) is a rare entity that either results from traumatic injury or can be spontaneously preceded or not by a minor trauma such as sporting activities. It represents a major cause of stroke in young patients. The diagnosis should be suspected with the combination of Horner's syndrome, headache or neck pain, and retinal or cerebral ischaemia. The confirmation is frequently made with a magnetic resonance angiography (MRA). Although anticoagulation with heparin followed by vitamin-K-antagonists is the most common treatment, there is no difference in efficacy of antiplatelet and anticoagulant drugs at preventing stroke and death in patients with symptomatic carotid dissection. We describe a patient with ICAD following deep sea scuba diving, who presented with Horner's syndrome and neck pain and was successfully treated with anticoagulants.

## 1. Introduction

Internalcarotid artery dissection (ICAD) is an uncommon entity, yet it is one of the major causes of cerebrovascular accident in patients younger than 45 years of age, accounting for 10–20% of ischaemic stroke cases. The annual incidence of ICAD is reported to be 2.6–2.9 per 100.000 [1]. The mean age is between 39 and 45 years. Males and females have equal occurrence rates, although females tend to be 5 years younger at the time of diagnosis.

ICAD can occur intracranially or extracranially, with the last one being more frequent.

The etiology [1–3] of ICAD can be spontaneous or traumatic. So-called traumatic ICAD are associated with major blunt or penetrating trauma.

These traumatic mechanisms are neck hyperextension with rotation, direct blow to the neck, blunt intraoral trauma, or basilar skull fracture.

Spontaneous ICAD can be preceded by a minor trauma and influenced by an underlying arteriopathy.

Minor trauma involves hyperextension and rotation of the neck [4]. These movements may cause the initial injury to the artery. These potential precipitating mechanisms of ICAD may be as follows.

1. Sporting activities: diving, karate, judo, skiing, football, rugby, yoga, jogging, ice hockey, swimming, horse riding, golf, tennis, cycling, basketball, and so forth.
2. Recreational activities: roller coaster, bungee jumping, and so forth.
3. Medical interventions: intubation, inadvertent intraoperative laceration, percutaneous carotid angiography, bronchoscopy, chiropractic manipulation, postpartum period, and so forth.
4. Others: coughing, vomiting, sneezing, and so forth.

The underlying arteriopathy may be Marfan's syndrome, fibromuscular dysplasia, cystic medial necrosis, Ehler-Danlos syndrome type IV, accumulation of mucopolysaccharides, osteogenesis imperfecta, polycystic kidney disease, arteritis/vasculitis, and minor abnormalities of the internal elastic lamina [2].

A history of hypertension, smoking, and oral contraceptive use have been shown to be more prevalent in ICAD patients; however atherosclerosis is distinctly uncommon in patients with ICAD [4].

The main consequences of dissection are as follows.

1. Stretching or compression which causes pain, Horner's syndrome, or cranial nerve paralysis.
2. Retinal or cerebral embolisms.
3. Subarachnoid haemorrhage.

Diving-related ICAD may be explained with this pathophysiologic mechanism of motion-dependent wall injury. It is discussed if a gas embolism because of a rapid ascent is a possible cofactor [5, 6].

## 2. Case Presentation

A 32-year-old man was admitted to emergency area for left-cervical pain, starting minutes after deep scuba diving in the Mediterranean sea six days before. At the same time, the patient noticed a feeling of pressure in his left ear. There was not a rapid ascent to the water surface, which may have caused a decompression illness. The weight of the diving gear did not exceed the usual weight range for recreational diving. The sea was calm and the water temperature was about 21°C. The whole dive took 3 hours. He was an experienced diver.

That morning he presented an increasing pain in the upper anterolateral left-cervical region. He was treated with anti-inflammatories nonsteroidal the days before without improvement.

He was allergic to povidone-iodine and smoker of 4-5 cigarettes per day. He denied past medical problems and did not usually take any medications. He denied alcohol or other drug use.

The examination of the cranial nerve territory showed a myosis and ptosis ipsilateral. The rest of the neurological examination was normal. The haematological and biochemical profiles were normal.

The electrocardiography trace showed sinus rhythm at a rate of 56 bpm. The chest X-rays and computed tomography scan of the head and neck were both normal.

The patient was diagnosed with a painful left-sided, incomplete Horner's syndrome based on the anisocoria and ptosis. He was hospitalized to complete the study.

**Figure 1:**
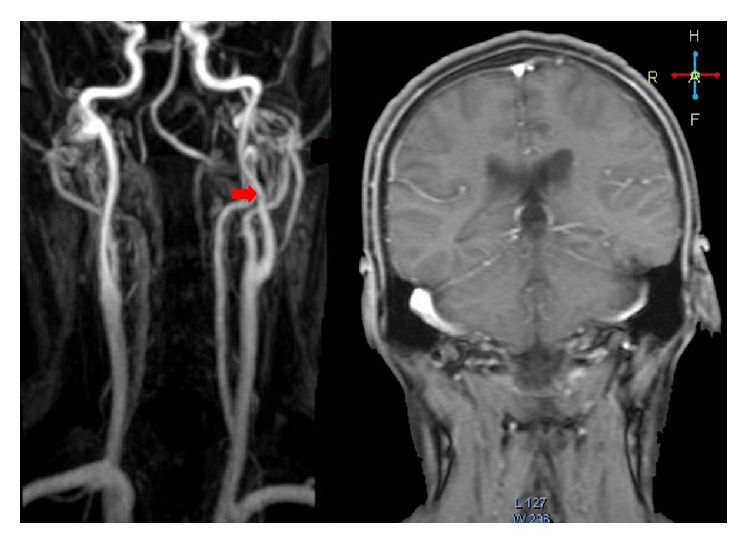
MRA and MRI with coronal slices demonstrating moderate narrowing of the left internal carotid artery just below the skull base (red arrow).

**Figure 2:**
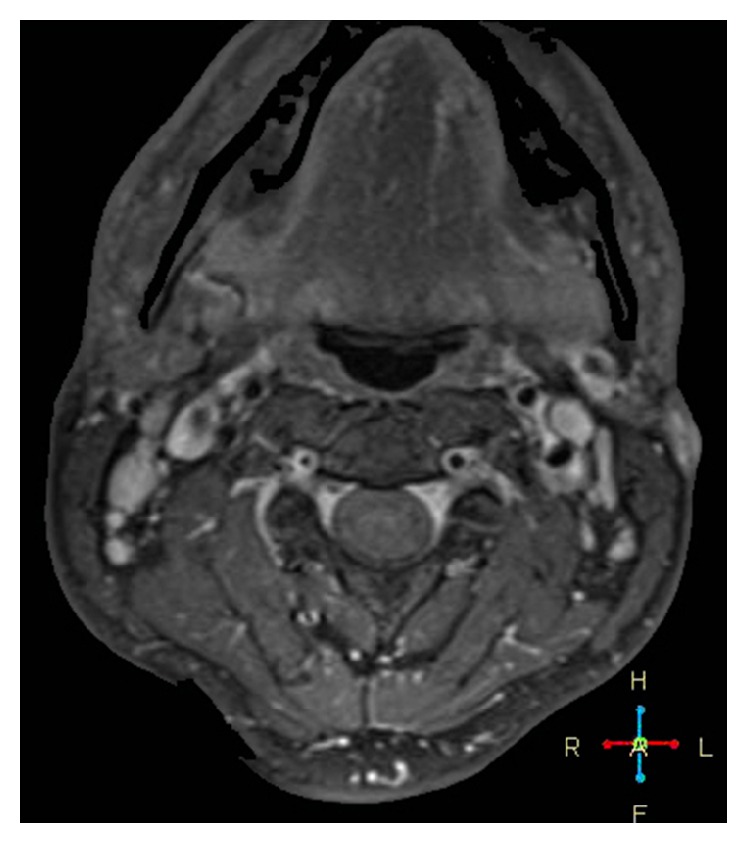
Axial source image of MRA performed just below the foramen lacerum showing the characteristic half-moon appearance of the false lumen separated by a thin flap from the oval-appearing true lumen.

A magnetic resonance image of the brain and MRA of the extra- and intracranial arteries were performed. It revealed a decrease in the calibre of the left ICA with an eccentric and irregular stenosis (string sign) (red arrow) at the base of the skull (Figure 1). The axial slices demonstrated the characteristic half-moon appearance of an internal carotid dissection of the left internal carotid artery (Figure 2).

A thrombophilia screen was requested including protein C, protein S, and lupus anticoagulant. This detailed screen did not reveal any abnormality.

The patient was started on intravenous heparin therapy for management of the ICAD followed by vitamin-K-antagonists (acenocoumarol) with an INR of 2.0–3.0 to prevent thromboembolism. After five days his clinical condition improved and he was discharged home. The ptosis and the cervical pain resolved after eight weeks. Out-patient follow-up at three months showed a minor myosis. The rest of the neurologic examination was normal. Repeat MRI examination at six months confirmed that the internal carotid artery had become patent with normal flow. After being anticoagulated for 6 months he started 100 mg of acetylsalicylic acid once daily.

## 3. Discussion

The neurological pathology after scuba diving has increased in the last years, because of its increased popularity. It can be associated with a dysbaric air embolism or decompression sickness. But it may be due to rarer causes as an ICAD [7].

The clinical manifestations [3] of the ICAD can be local symptoms and signs such as unilateral headache (periorbital and frontotemporal, facial, or anterior neck pain), Horner's syndrome (myosis, ptosis, and anhydrosis), and cranial nerve paralysis. Horner's syndrome is due to compression, stretching, or hypoperfusion of the sympathetic fibres within the carotid wall.

A painful Horner syndrome of acute onset is almost pathognomonic of carotid dissection. The hypoglossal nerve is most commonly affected, followed by cranial nerves IX, X, XI, and V. One possible mechanism leading to cranial nerve palsy is compromise of the vasa nervorum. Direct compression of the cranial nerves by the mural haematoma is another possible explanation.

These local signs are followed a few hours or days later by signs and symptoms of retinal and cerebral ischaemia. These manifestations are amaurosis fugax, retinal infarction (rare), and stroke, mainly in the middle cerebral artery territory [1].

The presence of any 2 of this triad (pain of the head, face, or neck, Horner's syndrome, and retinal or cerebral ischaemia) should initiate an investigation for ICAD [3] as an emergency.

The gold standard for diagnosing ICAD is conventional arteriography, but MRA is replacing it. Computerized tomographic angiography is more available and may be used for the diagnosis, although there is less experience. Ultrasonography has been increasingly used in the diagnosis of ICAD [2].

Treatment for ICAD involves emergent anticoagulation or antiplatelet therapy.

The risk of early recurrence of stroke has led many clinicians to advocate the use of anticoagulation from presentation until 3 or 6 months after dissection. However previous studies, including a Cochrane systematic review, found no difference in efficacy of antiplatelet and anticoagulant drugs at preventing stroke and death in patients with spontaneous extracranial internal carotid artery dissection [8].

Anticoagulants might prevent embolism from a fresh thrombus but they are also more hazardous than antiplatelet drugs and can result in extension of the intramural haemorrhage, which occurs in a third of patients according to MRI [9, 10].

The consensus among authors recommends heparin followed by vitamin-K-antagonists with an internal normalized ratio (INR) of 2.0–3.0 during 3–6 months to prevent thromboembolism. In patients with large cerebral infarcts or infarcts with haemorrhagic transformation, they start with aspirin for 10 to 21 days depending on the size of the infarct and then change to anticoagulants for 3 to 6 months. In the case of subarachnoid haemorrhage no antithrombotic treatment is preferred. When the artery is normalized they must stop therapy, but when there is an underlying arterial disease they take aspirin 100 mg daily for long-term prevention [1, 4].

If medical treatment fails or is contraindicated, surgery via balloon dilatation and intravascular stents are considered to restore luminal diameter [1, 5].

The prognosis of ICAD is highly variable. It is excellent when it is diagnosed with local signs. Clinical functional outcome depends on the initial stroke severity. The mortality rate is less than 5%. 85–90% of patients will have complete resolution in 3–6 months, and headache resolves in 95% of individuals with appropriate therapy. Symptomatic recurrent ICAD is uncommon (2% during the first month) and mainly occurs in a different artery [1, 4].

## Abbreviations

ICAD: Internal carotid artery dissection
INR: Internal normalized ratio
MRA: Magnetic resonance angiography
MRI: Magnetic resonance imaging.
